# The Royal National Sanatorium

**Published:** 1914-07-25

**Authors:** A. G. A. Major

**Affiliations:** Secretary


*To the Editor of* The Hospital.

### The Royal National Sanatorium.

*To the Editor of* The Hospital.

Sir,—I extremely regret to find that in your issue of to-day it is reported that the salaries of the nursing staff of this institution have been reduced. This is exactly the opposite of what has happened. If I stated otherwise in my communication to you, it is an error on my part and could only have arisen through putting the new and the old scales under the wrong headings. The following is the correct statement of the alterations made by my committee, and I shall be grateful if you will give the correction as prominent a position in your next issue as is given to the paragraph in the current number. If the error has arisen through my fault, I offer you my sincere apology for the trouble I am causing you: (1) Nursing staff and salaries *as formerly*; three sisters, commencing at £35, rising to £40, with £5 extra in lieu of uniform; seven probationers, at £10 for first year, £12 for second year, £15 for third year, uniform provided. (2) *New scale*—Present staff and salaries: one senior sister, £38 rising to £46, with £5 extra in lieu of uniform; two junior sisters, £36, rising to £40, with £5 extra in lieu of uniform; two staff nurses, at £28, rising to £34, uniform provided; five probationers, at £12 first year, £15 second year, £18 third year, uniform provided.

Yours faithfully,

July 18. A. G. A. Major, Secretary.

[We are very pleased to give publicity to this letter and to observe that the illiberal policy on which we commented has not in fact been adopted. As Mr. Major states, the mistake was his, not ours, as the information on which we relied was contained in his own letter: he did, in fact, place the wrong headings to the two columns, showing the old and new arrangements respectively.—Ed. The Hospital.]

